# Functional Properties and Safety Considerations of Zinc Oxide Nanoparticles Under Varying Conditions

**DOI:** 10.3390/nano15120892

**Published:** 2025-06-10

**Authors:** Ana Rita Mendes, Carlos M. Granadeiro, Andreia Leite, Otmar Geiss, Ivana Bianchi, Jessica Ponti, Dora Mehn, Eulália Pereira, Paula Teixeira, Fátima Poças

**Affiliations:** 1 Universidade Católica Portuguesa, CBQF—Centro de Biotecnologia e Química Fina—Laboratório Associado, Escola Superior de Biotecnologia, Rua Diogo Botelho 1327, 4169-005 Porto, Portugal; s-anrimamendes@ucp.pt (A.R.M.); pcteixeira@ucp.pt (P.T.); 2 REQUIMTE/LAQV & Department of Chemistry and Biochemistry, Faculty of Sciences, University of Porto, 4169-007 Porto, Portugal; cgranadeiro@fc.up.pt (C.M.G.); acleite@fc.up.pt (A.L.); eulalia.pereira@fc.up.pt (E.P.); 3 European Commission, Joint Research Centre (JRC), 21027 Ispra, Italy; otmar.geiss@ec.europa.eu (O.G.); ivana.bianchi@ec.europa.eu (I.B.); jessica.ponti@ec.europa.eu (J.P.); dora.mehn@ec.europa.eu (D.M.); 4 CINATE—Laboratório de Análises e Ensaios a Alimentos e Embalagens, Escola Superior de Biotecnologia, Universidade Católica Portuguesa, Rua Diogo Botelho 1327, 4169-005 Porto, Portugal

**Keywords:** ZnO nanostructures, morphology and size, functional properties, antibacterial efficacy, temperature effect, ROS generation, food safety

## Abstract

Zinc oxide nanoparticles (ZnO NPs) exhibit diverse morphologies and sizes, influencing their functional properties. However, the relationship between their morphology and behavior under varying conditions remains poorly understood. This study provides novel insights by linking ZnO NPs shape to generation of reactive oxygen species (ROS), and to antimicrobial efficacy under varying temperatures. ROS generation was confirmed via electron paramagnetic resonance, although no antioxidant activity was observed. Antibacterial tests against *Escherichia coli* and *Staphylococcus aureus* at different temperatures (4–22 °C) revealed that sheet-shaped NPs achieved complete bacterial reduction (7.5 log CFU mL^−1^ for *E. coli* at 4 and 22 °C; 6.8 log CFU mL^−1^ for *S. aureus* at 22 °C). Flower-shaped NPs were less effective due to larger size and reduced surface area. Zeta potential ranged from −44 to −58 mV, indicating high stability, with sheet-shaped particles being the most dispersed. Scanning electron microscopy confirmed closer interaction between sheet-shaped NPs and *E. coli* in agreement with the higher activity. Antibacterial efficacy decreased at 4 °C, highlighting implications for cold storage. The Weibull model successfully described *E. coli* reduction. These aspects were not previously addressed in the published work. The effect of temperature on the activity and its modeling are new insights into the morphology-dependent antimicrobial activity of ZnO NPs, supporting their integration into packaging materials for food applications.

## 1. Introduction

Nanotechnology is an interdisciplinary field with a wide range of commercial and scientific applications, focusing on materials with at least one dimension below 100 nm [1]. Nanomaterials exhibit enhanced functional and physicochemical properties compared to bulk materials due to their small size and high specific surface area [2]. The use of nanoparticles (NPs) is steadily increasing, driven by significant advancements in industries, such as electronics, medicine, food engineering, agriculture, energy, cosmetics, and telecommunications [3]. Among these, metal oxide NPs, such as titanium dioxide, magnesium oxide, and zinc oxide (ZnO) have attracted particular attention, leading to their integration into various technological and industrial applications [4].

ZnO NPs are among the most widely used metal oxides due to their diverse morphologies, sizes, and high surface-to-volume ratio, which directly influence their physicochemical properties. These characteristics contribute to their antibacterial, photocatalytic, and oxygen-scavenging properties, enabling applications in food preservation and safety [5]. The incorporation of NPs into polymeric materials has attracted significant attention in food packaging, enhancing mechanical strength, functionality, and barrier properties. These advanced packaging systems are classified as active and intelligent, ensuring protection, preservation, and extended food shelf life [6,7,8]. The use of ZnO NPs as antimicrobial agent can contribute to sustainability by extending products shelf life, reducing food waste and potentially decreasing the reliance on chemical preservatives [9].

Microbial infectious diseases are an increasing health concern in both developing and developed countries. Foodborne pathogens, such as bacteria and viruses, contaminate food products resulting in foodborne infections. The growing concern over resistant microorganisms motivates the research into novel antimicrobial compounds [10]. The incorporation of antimicrobial NPs into food packaging materials is an approach to inhibit and control microbial growth, ensure safety and extend product shelf life [11].

Several studies have demonstrated the capacity of ZnO NPs to reduce, inhibit, or retard the spoilage and pathogenic microorganism growth [12,13]. The nanometric properties linked to high specific surface area, enable these particles to infiltrate and interact with the bacterial cell wall, exhibiting great toxicity effect. In fact, the effectiveness of ZnO NPs is highly dependent on their morphology, size, specific surface area, applied dosage and exposure time to the bacteria cells [14].

Despite the large number of studies, understanding of the functional properties and behavior of ZnO NPs under varying conditions remains limited. In particular, the effect of temperature has not been adequately addressed, as most studies have been conducted in optimum laboratory conditions instead of those observed in real use scenarios. This study uniquely addresses this gap by systematically evaluating the antibacterial activity and functional properties of ZnO NPs with distinct morphologies at different storage temperatures, providing practical insights for their potential use in food packaging systems. The ability of ZnO NPs to generate reactive oxygen species (ROS) and their antioxidant activity were assessed. The interface between particles and the *E. coli* cells was analyzed. Viable cell count assays were performed towards *E. coli* (Gram-negative bacteria) and *S. aureus* (Gram-positive bacteria) at different temperatures and the Weibull model was used to describe the effect of temperature in the antimicrobial properties.

## 2. Materials and Methods

### 2.1. Zinc Oxide Nanoparticles

ZnO NPs were synthesized via different sol–gel methods as previously described in our earlier work [15], aiming to obtain three different morphologies. A short summary of the process is following described.

For spherical shape (ZnO-SP) synthesis, zinc acetate dihydrate (Sigma-Aldrich, Darmstadt, Germany), ethanol (Valente & Ribeiro, Alcanena, Portugal) and oxalic acid dihydrate (E. Merck, Darmstadt, Germany) were employed under high-temperature conditions. For flower shape (ZnO-FL) procedure, zinc nitrate hexahydrate (Sigma-Aldrich, Darmstadt, Germany) was dissolved in distilled water and reacted with sodium hydroxide (Fisher Chemical, Brussels, Belgium), under thermal conditions. For sheet shape (ZnO-SH), similar to ZnO-SP, zinc acetate dihydrate was used as a precursor but mixed with distilled water, followed by sonication with sodium hydroxide at room temperature.

### 2.2. Characterization of ZnO NPs

#### 2.2.1. Transmission Electron Microscopy

Transmission electron microscopy (JEOL-JEM 2100, JEOL, Milan, Italy; working at 120 kV) was used to verify morphology and primary particle size of each ZnO NPs stock suspensions. In particular, 1 mg mL^−1^ stock suspensions were prepared in ultrapure water, sonicated for 15 min by a vial tweeter (VialTweeter am UIS250v, Hielscher Ultrasonics GmbH, Teltow, Germany) at 75% amplitude, 0.5 cycle, and diluted to 0.1 mg mL^−1^ in ultrapure water. Each suspension (3 µL) was manually deposited on Formvar carbon-coated 200 mesh copper grids (Agar Scientific, London, UK), pre-treated by glow discharge (EM ACE600; 10 mV, 30 s, Leica, Buccinasco, Italy), and left to dry overnight in a desiccator, and images were acquired by transmission electron microscopy (TEM) at 120 kV. The primary particles size distribution was measured manually using ImageJ software (version 1.8.0), counting at least 100 particles. The results are reported as average of min Feret for one-dimensional ZnO-SP, Feret and min Feret for two-dimensional ZnO-SH, and length and width for two-dimensional ZnO-FL (nm).

#### 2.2.2. Dynamic Light Scattering

The intensity weighted mean hydrodynamic size (z-average) of the three types of tested ZnO NPs was determined using dynamic light scattering (DLS). Zinc oxide suspensions of 100 ppm were prepared in ultrapure water, bath-sonicated for 10 min, and vortexed immediately before transferring exactly 1 mL into the cuvette. Three measurements were taken for each sample using a Zetasizer Nano-ZS (Malvern Panalytical Ltd., Malvern, UK) DLS instrument. The measurement position, depth, and attenuator were automatically optimized as part of the DLS parameters, and the dispersant viscosity was set at 0.9308 mPa·s (water at 23 °C). The general-purpose analysis model was applied. The results represent averages from three measurements.

### 2.3. Functional Properties of ZnO NPs

#### 2.3.1. Generation of Reactive Oxygen Species

To assess the capacity for producing ROS, electron paramagnetic resonance (EPR) spectra were obtained using a Bruker ELEXSYS E500 spectrometer operating at 9 GHz (X-band) under the following experimental conditions: modulation frequency of 100 kHz, microwave power of 20 mW, modulation amplitude of 1 G, 60 dB of receiver gain, acquisition time of 300 s, and five scans. The spin trap experiments were performed by mixing 5 mg of the ZnO NPs with 20 µL BMPO (5-tert-butoxycarbonyl-5-methyl-1-pyrroline-N-oxide) solution (0.058 M) and 180 µL of Ringer’s solution. Aliquots from the reacting mixture were collected, transferred into a capillary, and placed in quartz EPR tubes. Samples were analyzed at room temperature. Spin Hamiltonian parameters were obtained using the computer program Bruker WinEPR/SimFonia (Version 1.2).

#### 2.3.2. Antioxidant Activity

The antioxidant activity was determined using methods based in 2,2-azino-bis-3-ethylbenzothiazoline-6-sulphonic acid (ABTS) and 2,2-diphenyl-1-picrylhydrazyl (DPPH).

##### ABTS

The ABTS assay was performed according to the procedure described by Ozgen et al., 2006 [16]. The stock solution was prepared by mixing these two solutions: 0.19 g of ABTS (Sigma-Aldrich, Darmstadt, Germany) in 50 mL of water (concentration of 7 mM) and 0.03 g of potassium persulfate (Merck, Darmstadt, Germany) in 50 mL of water (concentration of 2.45 mM). The stock solution was stored in the dark at room temperature for 16 h to reach a stable oxidative state before use. At 734 nm, the ABTS work solution’s initial absorbance was 0.700 ± 0.020 after being corrected with water. Briefly, 10 µL of sample was allowed to react with 1 mL of ABTS work solution in dark conditions and room temperature, and the absorbance was read at 734 nm after exactly 6 min (Shimadzu UV mini-1240 spectrophotometer, Kyoto, Japan). A blank was taken with distilled water. A calibration curve was performed with ascorbic acid equivalent antioxidant capacity (AAEAC) (Sigma-Aldrich, Darmstadt, Germany). A set of standard solutions of ascorbic acid were prepared by dilution of the stock solution. Three replicates were analyzed.

##### DPPH

The DPPH assay was carried out in accordance with the protocol outlined by Alexandre et al., 2019 [17], with a few adjustments. To prepare the stock solution (600 µM), 24 mg of DPPH (Sigma-Aldrich, Darmstadt, Germany) was dissolved in 100 mL of methanol (Fisher Scientific, Lisbon, Portugal). The solution was then kept at −20 °C in the dark. The DPPH work solution was adjusted with methanol to an initial absorbance of 0.700 ± 0.02 at 515 nm. Briefly, 25 µL of the sample was placed on a 96-well microplate (Nunc™, Thermo Fisher Scientific Inc., Waltham, MA, USA) and allowed to react with the DPPH work solution (175 µL) for 30 min at room temperature in the dark. The microplate was then read at 515 nm. A blank was taken with methanol. A calibration curve was performed with Trolox (Sigma-Aldrich, Darmstadt, Germany), and a set of standard solutions were prepared by dilution of the stock solution. The analyses were performed in triplicate.

#### 2.3.3. Antibacterial Activity

The antibacterial activity of the ZnO NPs was investigated by viable cell count assay toward two bacterial strains: *E. coli* ATCC 29215 (Gram-negative) and *S. aureus* ATCC 6538 (Gram-positive). A bacterial population of 10^8^–10^9^ CFU mL^−1^ was obtained by aseptically inoculating test microorganisms in brain heart infusion (BHI) broth and then incubating them for 16 h at 37 °C. After being centrifugated at 7000 rpm/8 min, the cells were resuspended in Ringer’s solution. The necessary quantity of ZnO NPs was added to the inoculum to prepare a 100 mg mL^−1^ suspension of each, which was then incubated at 4 and 22 °C. Additionally to these temperatures, the ZnO-SH was also incubated with the *E. coli* inoculum at 10 °C. Samples were obtained at 4, 24, 48, and 168 h, based on preliminary studies, to assess microbial reduction dynamics. Considering the potential for food storage up to one week, the duration was extended to verify the reduction in microorganisms to a level still detectable within the method’s detection limit (referred to as “total reduction” in this study).

The bacterial survival was assessed by plating appropriately diluted samples on BHI agar. Bacterial suspensions in Ringer’s solution without particles served as controls. The results represent an average of three separate replicates. The loss of cell viability was determined as follows:

(1) $$R\left(t\right)=Log\left(\frac{N}{N_{o}}\right)$$

where “R(t)” represents reduction over time, “N” refers to the CFU mL^−1^ after exposure for a specific time, and “N_0_” corresponds to the initial level (CFU mL^−1^) before exposure.

The kinetics of *E. coli* reduction in contact with ZnO-SH at 4, 10, and 22 °C were analyzed by mathematical modeling using the Weibull Equation (2). This model can represent a curve with an exponential pattern and has many applications in studies regarding technologies and industrial treatments [18]. This method is an alternative to the linear model and can be used to describe convex, concave, and linear survival curves. The survival curves present two parameters (δ and p), typical of the Weibull distribution.

(2) $$\ln\frac{N}{N_{0}}={\left(-\frac{t}{\delta }\right)}^{p}$$

where “N” corresponds to the CFU mL^−1^ after exposure for a certain time, “N_0_” corresponds the initial level (CFU mL^−1^) before exposure to NPs, t is the time, δ is the time for the first decimal reduction, and p value refers to the shape of the curve (p < 1 for convex curves and p > 1 for concave curves) [19,20].

#### 2.3.4. Interface *E. coli*/ZnO NPs and Stability of Particles

Scanning electron microscopy (SEM) analyses were performed to assess the interface between the ZnO NPs of different morphology and the cell wall of *E. coli* when brought into contact. A total of 15 mg mL^−1^ of ZnO NPs was prepared by adding the required amount of nanoparticles to the *E. coli* inoculum and incubated at 22 °C. Samples were collected after 4 days.

For the preparation of ZnO NPs/*E. coli* for SEM, tissue culture coverslips of 13 mm (SARSTEDT, Nümbrecht, Germany) were covered by poly-L-lysine solution (Agar Scientific, Rotherham, UK) and incubated at 37 °C for 1 h. The poly-L-lysine was removed, and the coverslips were again incubated at 37 °C for 1 h until complete drying. The ZnO NPs with *E. coli* samples were added and kept at 22 °C overnight. The samples were fixed with 2.5% glutaraldehyde (Sigma-Aldrich, Darmstadt, Germany, Germany) and washed with phosphate buffer 0.1 M. Samples were dehydrated with graded ethanol series (10, 30, 50, 70, 80, 90, and 100%), with 10 min treatment for each ethanol concentration and were then incubated in a vacuum oven for 1 h, 40 °C, and 600 mbar. SEM analyses were performed using a FEI QUANTA 400 FEG ESEM (FEI, Hillsboro, OR, USA) microscope equipment, operating at an accelerating voltage of 15 kV.

To complement the SEM analysis and assess the stability and surface properties of the ZnO NPs, zeta potential measurements were performed. Zeta potential was determined with a Zetasizer Nano ZS instrument (Malvern Instruments, Worcestershire, UK) equipped with a 633 nm laser and using a disposable zeta potential cell. The suspension was prepared by dispersing 10 ppm of ZnO NPs in 10 mM phosphate buffer (pH 7.0) and bath-sonicating it for 10 min. The measurement cell was rinsed with ethanol, with filtered distilled water and then loaded with the sample suspension. Measurements were taken at 25 °C with automatic reading and general data analysis settings using the Zetasizer v7.12 software. The results represent the average of three measurements.

### 2.4. Data Handling and Statistical Analysis

GraphPad Prism (version 8.4.2) and IBM SPSS Statistics (version 28) were used for statistical analysis. The results are expressed as average with corresponding standard deviations. To determine the differences between repeated measurements of various morphologies in antibacterial activity, linear mixed-effects models were employed. For pairwise comparisons of more than two means, Tukey’s post hoc analysis and one-way ANOVA were applied to find differences between each individual period. A statistically significant result was defined as a two-sided p < 0.05.

Weibull model parameters were estimated using IBM SPSS Statistics by nonlinear regression in order to minimize the sum of the squares of the differences between the predicted and experimental values. The regression quality was assessed by residual sum of squares (RSS) and coefficient of determination (R^2^).

## 3. Results and Discussion

### 3.1. Size and Morphology of ZnO NPs

**Figure 1:**
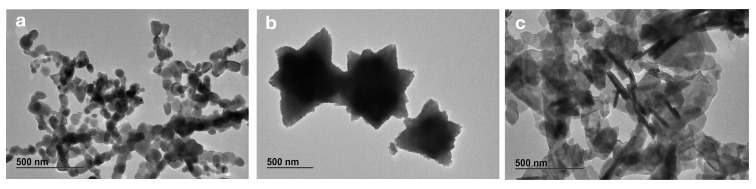
TEM micrographs of ZnO NPs: ZnO-SP (**a**), ZnO-FL (**b**), and ZnO-SH (**c**). Magnification: 15,000×.

In previous studies, size and morphology of the ZnO NPs were determined by SEM [15]. However, measurements using TEM can provide a more detailed visualization of the particles. TEM micrographs confirmed the synthesis of ZnO NPs with spherical, flower, and sheet shapes (Figure 1a–c). By manual measurement of the particle size, it was possible to conclude that the ZnO-SP has a one-dimensional min Feret size of 45 ± 13 nm. Regarding ZnO-FL, the min Feret is 1004 ± 261 nm but considering that the flower shape is an agglomeration of two-dimensional petals, the average petal length and width is 440 ± 107 nm and 304 ± 72 nm, respectively. The ZnO-SH is considered also as a two-dimensional nanoparticle and the min Feret size is 122 ± 73 nm and the Feret is 275 ± 140 nm. The size measurements by TEM are slightly higher but in the same value ranges of the manually SEM determination previously reported [15].

**Figure 2:**
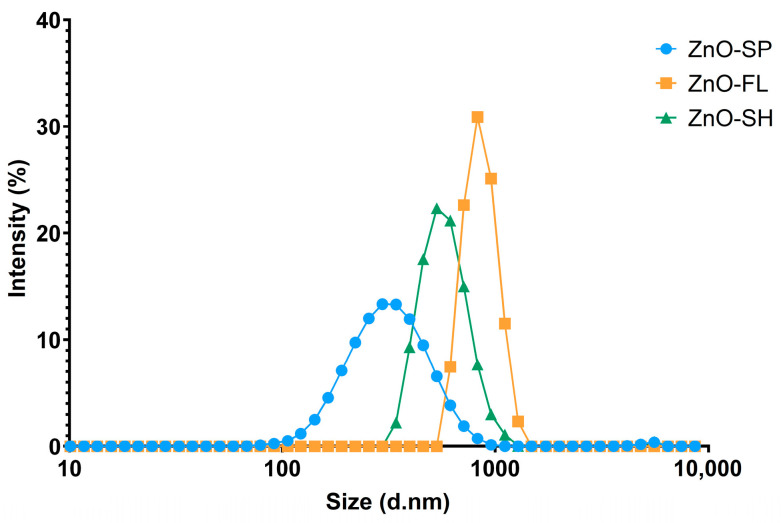
Intensity based size-distribution of ZnO-SP, ZnO-FL, and ZnO-SH (hydrodynamic diameter of equivalent spheres), obtained by DLS.

DLS measurements were used to determine the hydrodynamic diameter, polydispersity index (PDI), and size distribution of ZnO NPs in aqueous suspension, providing insights into their dispersion behavior and potential aggregation state under experimental conditions. The particle size distributions obtained, as depicted in Figure 2, show an average particle size in the following order: ZnO-SP (322 ± 10 nm) < ZnO-SH (802 ± 54 nm) < ZnO-FL (1182 ± 54 nm). The intensity weighted mean hydrodynamic size of the ensemble collection of particles (z-average) cannot be directly compared with the min Feret determined with TEM [21,22]. In fact, DLS measures agglomerates and aggregates as single large particles and is not capable of distinguishing and measuring constituent particles within these structures. Additionally, the data analysis of DLS are based on the assumption that the particles are uniform and spherical, which is not the case for the flower- and sheet-shaped ZnO NPs examined in this study.

The PDI is a parameter used to represent the broadness of a particle size distribution and is determined together with the z-average during DLS measurements [23]. The ZnO-SP showed the lowest PDI (0.29 ± 0.04), indicating an almost monodisperse distribution, followed by ZnO-FL and ZnO-SH with PDI of 0.38 ± 0.01 and 0.44 ± 0.05, respectively, representing moderately polydisperse size distributions [24]. Similar PDI values of 0.3 ± 0.0 and 0.4 ± 0.4 have been reported in the literature for ZnO NPs of 50 and 100 nm, respectively [25]. However, that work did not determine the PDI for different morphologies. In contrast, the ZnO nanostructures evaluated in this work have been thoroughly characterized in terms of their structural and physicochemical properties [15].

This characterization serves as a robust basis for the functional evaluation carried out in the present work. Such a combined and sequential approach remains underexplored in the literature, where studies often lack comprehensive characterization and seldom explore the relationship between nanoparticle shape and antibacterial performance.

### 3.2. Functional Properties

#### 3.2.1. Generation of Reactive Oxygen Species

To assess the potential ROS generation, ZnO NP suspensions in Ringer’s solution were prepared, and BMPO was used as a spin trap to record EPR spectra. The choice of this spin trap over the commonly used DMPO was driven by its superoxide radical adduct’s prolonged lifetime that far exceeds that of the DMPO adduct [26]. Additionally, it is noteworthy that the superoxide-DMPO adduct undergoes spontaneous decay, leading to the generation of the DMPO-hydroxyl adduct and this transformation leads to misleading interpretations [27].

**Figure 3:**
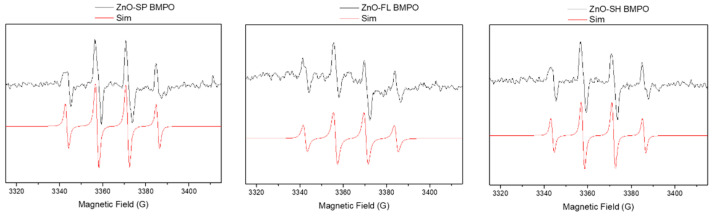
Room temperature EPR spectra of the ZnO NPs spherical, flower, and sheet, in Ringer’s solution in the presence of the BMPO spin trap. EPR spectrum (black line) and spectrum simulation (red line).

A characteristic BMPO-adduct giving rise to four resolved peaks was obtained for all ZnO NPs samples (Figure 3). It was clearly observed a four-line spectrum with relative intensities of 1:2:2:1, corresponding to the interaction of the unpaired electron with the nitrogen and hydrogen atoms, typical for the adduct formed between BMPO and the hydroxyl radicals (BMPO/OH). The isotropic Spin-Hamiltonian parameters obtained by simulation are presented in Table S2 and are in good agreement with the published data for the hydroxyl-BMPO adduct [27]. A control experiment was performed for BMPO in Ringer’s solution in the absence of ZnO NPs, and no EPR signal was detected.

The surface oxygen vacancies of the particles already reported [15] can explain the generation of ROS observed in the present study. These vacancies serve as active sites for the adsorption of molecular oxygen, which can then undergo reduction to form superoxide radicals [28]. However, no significant differences were observed between the three different morphologies of ZnO NPs. This suggests that the ROS generation might be predominantly driven by the overall density of oxygen vacancies rather than the specific shape of the nanoparticle.

#### 3.2.2. Antioxidant Activity

ZnO NP samples tested at different concentrations, including 0.5 and 1 mg mL^−1^ for ABTS and 1, 10, and 50 mg mL^−1^ for DPPH did not exhibit antioxidant activity, as the values of AAEAC and Trolox were negative or zero at the blank level (Tables S3 and S4). Therefore, it can be concluded that these synthesized NPs do not exhibit antioxidant activity. Many studies in the literature report antioxidant activity, when the ZnO NPs were synthesized using extracts from plants, fruits, leaves, or flowers [29,30,31]. A study revealed that commercial ZnO NPs exhibited no antioxidant activity compared to ZnO NPs derived from biosynthesis with the polyphenol-enriched fraction of pomegranate peel [32]. In fact, the functional groups present in the extracts are responsible for the antioxidant capacity of ZnO NPs (green synthesis approach). The use of natural resources as a stabilizing agent enables the ZnO NPs to show antioxidant activity due to the presence of phytochemical compounds such as phenols, alkaloids, and flavonoids, which are capped on the nanoparticle surface [33].

The absence of antioxidant activity in these ZnO NPs is likely due to the lack of surface functionalization, which limits their electron-donating capacity (essential for radical scavenging activity in assays like ABTS and DPPH). For instance, a study reported a fabrication of ZnO NPs functionalized with gallic acid that exhibit significantly enhanced antioxidant activity, emphasizing the importance of surface chemistry [34].

Despite not exhibiting inherent antioxidant activity, ZnO NPs are well-recognized for their ability to absorb and block UV radiation. Therefore, the beneficial effects observed in foods packaged in materials containing ZnO NPs with UV shielding should not be mistakenly attributed to antioxidant properties of the particles [35,36].

#### 3.2.3. Antibacterial Activity and Effect of Temperature

The antibacterial activity of different shapes and sizes of ZnO NPs was investigated by viable cell count assay of *E. coli* and *S. aureus* at 22 °C and 4 °C (Table 1). Prior studies have suggested that the antimicrobial activity of ZnO NPs is influenced by their morphology, size, and specific surface area [37,38].

The antibacterial performance of the three ZnO NPs morphologies was different when in contact with both bacteria at different temperatures. Significant differences (p < 0.001) between ZnO-SP and ZnO-SH at 24 h were recognized for *E. coli* at 22 °C.

A complete bacterial reduction was observed after 24 h when in contact with ZnO-SH (7.53 log reduction), and the values were statistically significantly lower than those of the other shapes (p < 0.05). The ZnO-SP also induced a total reduction in these bacteria but just after one week. Conversely, ZnO-FL did not reduce *E. coli* at 24 h when compared to the control, (p = 0.819), but a slight reduction of 4.63 log was achieved after one week.

Regarding the experiments at 4 °C and compared to the other morphologies, only ZnO-SH presented a total reduction in *E. coli* and just after one week (p < 0.001). When exposed to ZnO-SP and ZnO-FL, the bacteria demonstrated a very low reduction (1.34 and 0.84 log reduction, respectively) but with no statistical significance when compared with the control (p = 0.474 vs. p = 0.999, respectively).

The antibacterial results for *S. aureus* at 22 °C show that, similar to *E. coli*, ZnO-SP and ZnO-SH induced significant bacterial reduction compared to the control (p < 0.001) after 24 h, contrary to the ZnO-FL (p = 0.949). When in contact with ZnO-SP and ZnO-SH, *S. aureus* reduced completely after one week (6.83 log reduction), with moderately accelerated reduction in the presence of ZnO-SP. The time and morphology significantly influenced the reduction (p < 0.001). Post hoc comparisons revealed that the spherical and sheet morphologies differ from the flower shape and control, with no differences between them, revealing that these two shapes present the best reduction. On the other hand, ZnO-FL revealed lower bacteria decrease (3.71 log reduction) after one week.

At 4 °C, a slight reduction in *S. aureus* was observed among all the shapes. ZnO-SH showed an average reduction of 1.85 log, followed by 1.37 log reduction for ZnO-SP and 0.53 log reduction for ZnO-FL.

As a conclusion, ZnO-SH presented the best antibacterial performance among the tested shapes. When both bacteria were exposed to ZnO-SH at different temperatures, a total bacterial reduction was obtained (except for *S. aureus* at 4 °C, although still showing the best result compared to the other shapes). ZnO-SP also demonstrated to have an effective antibacterial activity since they induce total reduction in both microorganisms at 22 °C. Conversely, ZnO-FL presents the lowest antibacterial effect. In fact, this is in line with ZnO NPs physical properties, since ZnO-SH presented higher specific surface area (18.5 m^2^ g^−1^) [15]. The spherical shape exhibits the smallest size (41 nm) and a specific surface area of 13.4 m^2^ g^−1^. In contrast, ZnO-FL has the lowest surface area (5.3 m^2^ g^−1^) and the highest size (440 × 304 nm), resulting in a less efficient antibacterial effect. The findings obtained in this study align with previous studies, indicating that greater specific surface area and smaller sizes of NPs result in increased antibacterial activity because of the interactions with bacterial cells [38]. Despite these differences, all three ZnO NP morphologies exhibit antibacterial activity.

These findings highlight the potential of ZnO-SH (with superior performance) nanoparticles as a promising candidate for developing antibacterial food packaging, offering longer shelf life, and reduced microbial contamination during storage.

Few studies have previously explored the antibacterial activity of ZnO NPs with different morphologies and sizes [39]. Higher antibacterial activity against *E. coli* and *S. aureus* was observed for spherical ZnO NPs, compared to hexagonal and ellipsoidal nanoparticles, because of the lower size (30 nm) and the higher specific surface area (25.70 m^2^ g^−1^) of this morphology [40]. Despite the slightly higher specific surface area, these values are comparable to those found in this work. Another study synthesized three different types of flower-shaped ZnO NPs using controllable parameters and investigated the antibacterial activity against *S. aureus* and *E. coli* [41]. The authors found that the petal flower demonstrated higher antibacterial activity compared to fusiform flower and rod flower. In fact, the petal flower presents the highest surface area (7.21 m^2^ g^−1^), compared to the other flowers (2.72 m^2^ g^−1^ for fusiform flower and 3.28 m^2^ g^−1^ for rod flower). The ZnO-FL of this work is similar to the petal flower but with slightly lower specific surface area (5.3 m^2^ g^−1^). ZnO NPs with hexagonal, spherical, cylindrical, and cuboidal shapes were inspected for the antibacterial activity against *Bacillus subtilis*, *S. aureus*, and *E. coli* using the agar well diffusion method [42]. Compared to spherical (60–180 nm) and hexagonal-shaped NPs (63 nm), cuboidal-shaped NPs (40–45 nm) demonstrated more efficient microbial inhibition, justifying the influence of size in antibacterial activity. However, the particle surface area was not determined in this study.

In this study, *E. coli* was less resistant to ZnO NPs, compared to *S. aureus*. Previous studies have reported similar results, showing a more pronounced inhibitory effect of ZnO NPs on Gram-negative bacteria compared to Gram-positive bacteria [13,43]. In contrast, another study shows that *S. aureus* was more sensitive to ZnO powders than *E. coli* [40]. This study indicates that particle characteristics influence antibacterial efficiency differently in each bacteria: for *E. coli*, with its elongated shape, particle morphology plays a major role in antibacterial activity, whereas in *S. aureus*, which has a round shape, morphology is less significant, and the specific surface area of ZnO NPs is more critical to overall antibacterial effectiveness. Furthermore, the different susceptibility of the two bacteria to the ZnO NPs may be explained by their cell wall properties. Despite having a similar internal layer, both bacteria have very different external structures. Gram-positive bacteria have a thick peptidoglycan layer that contains teichoic and lipoteichoic acids. Gram-negative bacteria have a thin peptidoglycan layer with an outer membrane that contains lipopolysaccharide, phospholipids, and proteins. Even with this additional layer, the inactivation of bacteria by ZnO NPs activity is higher in Gram-negative bacteria [44,45].

The effect of temperature on the antibacterial activity of ZnO NPs can be observed for both bacteria. At 4 °C, the antibacterial activity is not as effective when compared to 22 °C, as expected. It has been reported that by increasing the temperature, the toxicity of ZnO NPs increases and, consequently, improves the antibacterial activity [46]. The temperature range is important and should simulate the conditions to which food may be exposed, from refrigeration to higher temperatures found in real environments. The NPs here characterized were designed to be incorporated into a matrix for application as food packaging. Therefore, the selected temperatures align with typical storage conditions and potential temperature variations encountered in food handling processes.

The Weibull kinetic model was fitted to the experimental data for microbial reduction in *E. coli* in the presence of ZnO-SH at 4, 10, and 22 °C. This model has been widely used to express the microbial inactivation [20], including the effect of NPs like zeolite nanoparticles in *E. coli* survival [47].

In our study, to investigate the kinetics, a single bacterial strain and one type of ZnO NP were selected. The morphology exhibiting the highest antibacterial activity (ZnO-SH) and the least resistant bacterium (*E. coli*) were chosen. The microbial inactivation parameters were estimated and are presented in Table 2 for all temperatures. The scale parameter, δ, represents time required for the first log-reduction. The lower the value of this parameter, the more effective the microbial inactivation [19].

**Figure 4:**
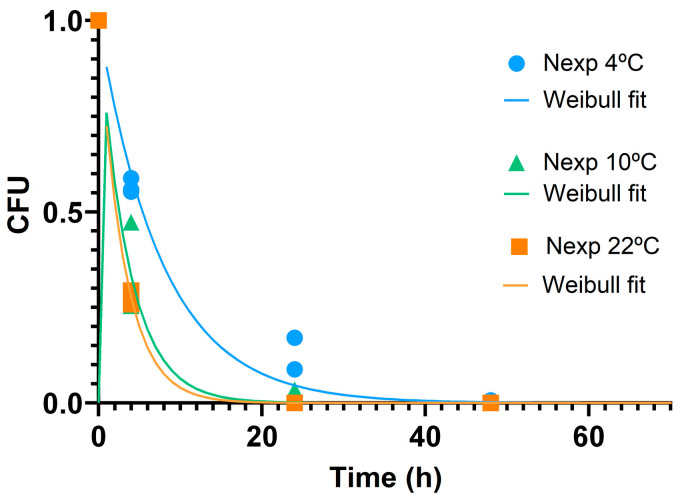
CFU representation of Weibull model for ZnO-SH at: 4 °C; 10 °C; 22 °C.

A decreasing trend in the δ value with an increase in temperature can be observed. However, to model the effect of temperature on this parameter, tests at additional temperatures should be conducted for more robust modeling. The parameter, p, indicates the shape of the curve and represents an effect of inactivation when it is less than 1 [19]. All the p value are <1, indicating the convex curves and the effective *E. coli* cell reduction at three different temperatures. The R^2^ are close to 1 in all cases which indicates that Weibull model is well fitted, so it has excellent ability to predict the CFU mL^−1^ along the time. The graphical representation of the experimental and predicted values for all the temperatures are represented in Figure 4.

Applying the Arrhenius model could be insightful, but additional data at a broader range of temperatures and over more frequent sampling time points would be necessary.

In this study, the modeling results for sheet-shaped NPs are reported, but expansion of the model for ZnO NPs with different shapes and sizes will be of interest. By applying this model, we can forecast the potential performance of ZnO nanoparticle-based packaging under real storage conditions, helping to optimize packaging designs and ensure consistent food safety standards across different temperatures.

#### 3.2.4. Interface E. coli/ZnO NPs and Particles Stability

**Figure 5:**
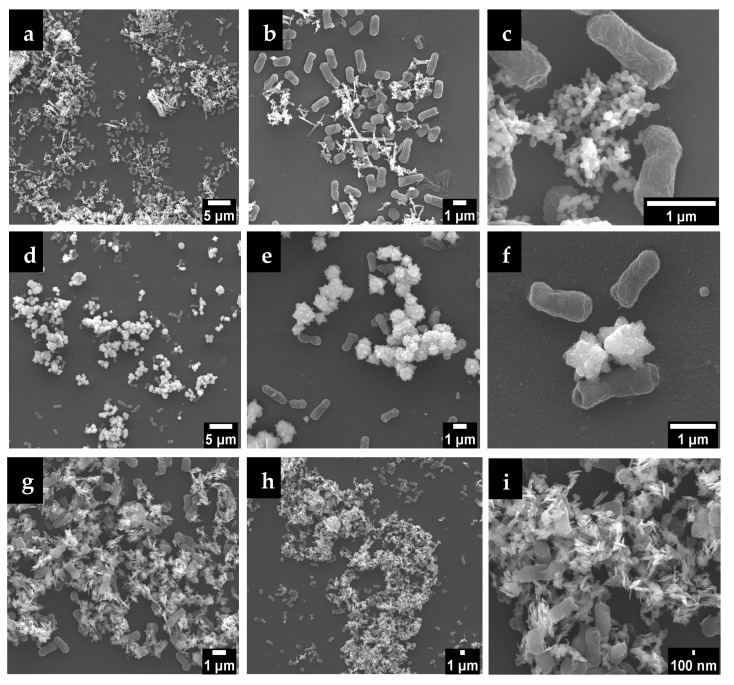
SEM micrographs of ZnO NPs in contact with *E. coli* after 4 days/22 °C incubation: ZnO-SP (**a**–**c**); ZnO-FL (**d**–**f**); ZnO-SH (**g**–**i**).

*E. coli* in contact with ZnO NPs suspensions were observed by SEM after 4 days at 22 °C (Figure 5). The morphologies of the ZnO NPs remained the same after the contact. In fact, the interaction of the different morphologies of ZnO NPs plays an important role in microbial cells. *E. coli* cells have an elongated structure with a rod-like morphology of approximately 1–3 µm of length. There is a tendency for the different nanoparticles to surround the bacteria as they are facing the membrane of the bacteria. *E. coli* bacteria are more likely to come into contact with a higher number of spherical and sheet particles, potentially explaining the enhanced antibacterial activity observed in this work.

As shown in Figure 5f, only the extremities of the petals of the flower-shaped NPs interact with the membrane of the bacteria. The contact area is smaller, and consequently the antibacterial activity is lower, when compared to the other shapes.

In contrast, smaller NPs can penetrate the bacterial membrane more easily due to the high interfacial area. A similar study also reports the tendency for NPs to surround the membrane of *E. coli* bacteria, but no systematic observations were presented regarding different particles shapes [48].

The exact mechanisms for the antibacterial action of ZnO NPs have not been fully elucidated [15]. Distinct mechanisms have been proposed in the literature for a better understanding of the ZnO NPs activity: electrostatic interactions by direct contact of the nanoparticles with cell walls, resulting in destruction of bacterial cell integrity; release of antimicrobial Zn^2+^ ions, that penetrate through the cell membrane, resulting in protein denaturation and loss of cell proliferation; formation of ROS that can cut off the chemical bonds of bacteria’s organic matter, causing destruction/penetration of the cell membrane [14,49].

**Figure 6:**
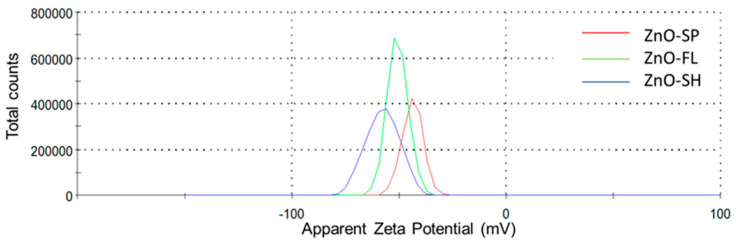
Zeta potential distribution of ZnO-SP, ZnO-FL, and ZnO-SH at pH = 7.

The electrostatic interactions are caused by the accumulation of positively charged Zn^2+^ on the microbial surface cell membrane, resulting in membrane dysfunction [49]. The zeta potential of the particles is a relevant measure of the electrostatic repulsion/attraction between particles and their dispersion stability, which in turn influences the interactions between ZnO NPs and microbial cell membranes. The potential difference between the particle surface and its surroundings arises from the presence of charged species at the interface [50]. Studies revealed that zeta potential depends on pH and conductivity of the dispersing medium, so different values are obtained depending on these parameters [51]. Generally, a higher magnitude of zeta potential (whether positive or negative) indicates better stability because the particles are less likely to aggregate [37]. In this work, at pH = 7, all particles are negatively charged with ZnO-SP presenting a peak at −43.7 mV, followed by ZnO-FL (−50.7 mV) and the lowest one (highest in absolute value), ZnO-SH with −57.5 mV (Figure 6 and Table S5). The ZnO NPs are strongly negatively charged and stable at neutral pH, exhibiting an electrostatic repulsion between the nanoparticles that provide stability in water-based suspensions. Although all ZnO NPs demonstrated good stability (<−30 mV), the sheet-shaped NPs exhibited the highest stability among the three morphologies. Similar studies also demonstrated negatively charged surfaces of ZnO NPs (i.e., negative zeta potential) but did not take into consideration different morphologies [52,53]. A previous study investigated the variation in zeta potential with different ZnO NPs shapes, demonstrating that pyramidal rod and star-like morphologies presented −33.2 and −11.8 mV, respectively, which is slightly lower (in absolute value) compared to the work here reported [54]. However, few studies have focused on the influence of ZnO NPs morphology on zeta potential. Therefore, it is of interest to explore these parameters in depth. Different pH levels can be tested to meet the requirements of the intended application. For instance, in food packaging applications, the experimental pH should be adjusted to simulate the conditions of the specific food model.

Bacterial cell membranes typically have a net negative charge due to the presence of lipopolysaccharides in Gram-negative or teichoic acids in Gram-positive bacteria. In this case, the ZnO NPs have a negative zeta potential, so there might be repulsion between the particles and the bacterial cell membranes, potentially reducing the efficiency of particle attachment and penetration [55]. However, higher magnitude (whether positive or negative) of zeta potential values generally indicates better dispersion and stability of the nanoparticles. Well-dispersed NPs have a larger surface area available to interact with bacterial cells, leading to an increased release of Zn^2^⁺, which can enhance antibacterial activity. This is in line with ZnO-SH properties that have the higher specific surface area and zeta potential (−57.5 mV) and, consequently, better antibacterial activity. Aggregated particles, on the other hand, have reduced surface area and might exhibit lower antibacterial efficacy. Furthermore, the amount of surface charge is morphology dependent and extended shape was proven to have more charge [56]. In fact, sheet-shaped NPs are the ones with flat/extended shape compared to the spherical and flower shape and, therefore, it is expected to have higher contact area with the bacteria. This can be demonstrated in Figure 5i, which shows the sheet shape with extended and sharp edges close to *E. coli*.

Zeta potential can also influence the production of ROS by ZnO NPs, since negatively charged ZnO NPs might interact differently with the environment compared to positively charged ones, affecting the generation and availability of ROS near bacterial cells. It was demonstrated that the three different shapes show capability of ROS formation (Figure 3).

Some authors believe that the release of ROS from the surface of ZnO is the major explanation for antibacterial activity, despite the effect of other factors that cannot be forgotten [57]. Hence, further research is required to fully elucidate the underlying mechanisms. The results indicate that the enhanced antibacterial activity of the ZnO NPs, particularly those with sheet morphology, is primarily due to a synergistic effect between ROS generation and increased surface interaction with bacterial membranes. The strong negative zeta potential contributes to particle stability and dispersion, promoting greater contact area and facilitating Zn^2^⁺ release.

## 4. Safety Considerations

The application of nanotechnology has grown considerably in recent years due to the unique physicochemical properties of nanomaterials. In addition to improving food safety, the use of ZnO NPs in packaging contributes to sustainability by extending products shelf life, reducing food waste, and potentially decreasing the reliance on chemical preservatives [9]. However, their nanoscale dimensions have raised concerns regarding safety. ZnO NPs may migrate from the packaging into food and, consequently, into human body. One of the major gaps in current research is the limited understanding of the long-term health effects of nanoparticles. Regulatory frameworks are not fully developed, as current food safety guidelines do not take into consideration the properties of nanoparticles [58].

One of the main mechanisms behind the antimicrobial efficacy of ZnO NPs, demonstrated in this work, is the generation of ROS. The release of hydroxyl radicals (·OH) and superoxide anions (·O_2_^−^) is closely linked to their antimicrobial properties, as these reactive species can disrupt microbial cell membranes and damage DNA [59,60]. While beneficial for microbial control, ROS may also pose risks to human health due to their potential to induce oxidative stress. For food packaging applications, it is, therefore, essential to evaluate the potential toxicity associated with ROS. The migration of ZnO NPs and, consequently, ROS from food packaging materials to food and, subsequently, into the human digestive system could cause oxidative stress, leading to cellular damage and contributing to the development of chronic diseases [61]. Safety assessments under regulatory standards should be conducted to ensure that ROS production and ZnO NPs migration do not exceed safe limits.

The absence of antioxidant activity observed in this study was disappointing. Future research may focus on the surface modification of ZnO NPs with natural antioxidants to combine the already proved UV-shielding with antioxidative properties for multifunctional food packaging materials.

The European Food Safety Authority (EFSA)’s guidelines emphasize the need for comprehensive safety assessments of nanomaterials intended for food contact applications. These include detailed data collection, physicochemical characterization of the NPs, migration studies, and toxicological evaluations. ZnO NPs have been considered safe, by EFSA, to be used in plastics packaging applications as ultraviolet (UV) light absorbers, but not at antimicrobial agents [62,63]. The evaluation relied on the fact that there is no significant migration in particulate form when particles are incorporated and fully embedded in plastic [62]. However, according to Regulation (EU) No 10/2011 and its amendment Regulation (EU) No 2016/1416 [64], the specific migration limit (SML) for zinc in food contact materials is set at 5 mg kg^−1^ of food. Migration behavior may vary depending on the packaging matrix and conditions of use, highlighting the need for further research to assess their safety and efficacy in different materials. In this context, recent studies have explored the integration of ZnO NPs into non-toxic natural polymer matrices, such as alginate, for food packaging applications. Alginate is water-soluble, biocompatible, and considered Generally Recognized As Safe (GRAS) by the US-FDA, which further supports its potential in safe and functional active packaging systems [65].

Evaluating the toxicity of nanomaterials requires a multifaceted approach that combines in vitro and in vivo, supported by advanced analytical methods to evaluate their potential health risks. In vitro studies are frequently employed to assess parameters, such as cytotoxicity, genotoxicity, and oxidative stress potential in cultured cells, providing preliminary insights into how nanomaterials interact with biological systems at the cellular level [61]. These evaluations are essential for identifying potential health risks and for defining safe exposure limits in food contact applications.

## 5. Conclusions

This study addresses the underexplored influence of morphology and size on the antibacterial activity of ZnO NPs, providing valuable insights for their application in the food packaging industry under different temperatures conditions. Functional and structural properties were studied across spherical, flower-, and sheet-shaped nanoparticles.

TEM analysis confirmed consistent morphologies and sizes of the synthesized ZnO NPs, aligning with previous work: spherical with 45 nm, flower with 440 × 304 nm, and sheet with 275 × 122 nm. Among the tested morphologies, sheet-shaped NPs demonstrated the highest antibacterial efficacy, achieving complete reduction in *E. coli* and *S. aureus* at 22 °C and of *E. coli* at 4 °C. In contrast, flower-shaped NPs showed the lowest activity, likely due to their larger size and limited surface interaction with bacterial membranes, as confirmed by SEM analysis. Notably, *E. coli* showed greater susceptibility than *S. aureus*, probably due to differences in membrane structure.

The antibacterial activity of ZnO NPs was found to be temperature-dependent, with higher efficacy observed at 22 °C. Furthermore, this work confirms that the Weibull model can fit as the microbial survival model to describe the behavior of *E. coli* when exposed to sheet-shaped ZnO NPs at 4, 10, and 22 °C.

ROS generation was confirmed for the three different ZnO NPs shapes, supporting its role as one of the possible antibacterial mechanisms. However, while ZnO NPs and the resulting ROS release are effective in combating bacteria, their presence in food packaging could also pose risks if they migrate into the food, potentially leading to oxidative stress in human cells upon consumption. Therefore, understanding the balance between the beneficial antimicrobial effects and the potential for toxicity is critical for the safe use of ZnO NPs. Zeta potential measurements revealed that all ZnO NPs were strongly negatively charged and stable at neutral pH, with sheet-shaped NPs showing the highest absolute zeta potential and dispersion stability. This dispersion contributes to their enhanced interaction with bacterial cell membranes, likely promoting ROS generation. Although the exact antibacterial mechanism is not yet fully elucidated, the results suggest that ROS-induced oxidative stress, in combination with increased surface interaction and Zn^2^⁺ ion release, plays a major role in the observed antibacterial activity.

These findings highlight the importance of nanostructure varieties in improving antibacterial properties and provide valuable insights for future applications of ZnO NPs as an antimicrobial agent to be further applied to food packaging industry. The significant variation in antibacterial activity among the different ZnO NPs morphologies indicates that the sheet-shaped ZnO NPs, in particular, may offer the best potential for enhancing food safety by inhibiting bacterial growth. Furthermore, this study demonstrates that higher temperatures significantly enhance the antibacterial activity of ZnO NPs, which aligns with the common practice of storing foods at refrigeration or slightly elevated temperatures. This knowledge can support the design of more effective food packaging solutions, reducing the risk of foodborne diseases and extending food shelf life. Potential safety concerns associated with oxidative stress and toxicity were also addressed, highlighting an important gap in current research.

For future research, it would be valuable to explore the interaction of ZnO NPs with different food matrices to better understand their behavior in real packaging scenarios. Investigating the long-term stability of these nanoparticles, the Zn migration rates, and potential toxicity when used in food contact materials is also crucial for ensuring consumer safety. Additionally, future studies could apply alternative synthesis methods, particularly green approaches, to further enhance the eco-friendly characteristics of these nanoparticles and their functional properties, including antioxidant activity.

## Figures and Tables

**Table 1:** Reduction in *E. coli* and *S. aureus* viability by the antibacterial activity of different ZnO NPs at 4 and 22 °C. Average ± standard deviation.

		R(t)							
		*E. coli*				*S. aureus*			
T (°C)	Time (h)	ZnO-SP	ZnO-FL	ZnO-SH	Control Bacteria	ZnO-SP	ZnO-FL	ZnO-SH	Control Bacteria
4	0	0	0	0	0	0	0	0	0
	4	−0.31 ± 0.17	−0.33 ± 0.03	−0.24 ± 0.09	−0.14 ± 0.07	−0.06 ± 0.39	−0.06 ± 0.22	−0.05 ± 0.11	−0.07 ± 0.04
	24	−0.63 ± 0.60	−0.51 ± 0.11	−0.95 ± 0.22	−0.32 ± 0.07	−0.33 ± 0.16	−0.07 ± 0.30	−0.35 ± 0.11	0.10 ± 0.24
	48	−0.46 ± 0.25	−0.66 ± 0.04	−2.30 ± 0.14	−0.55 ± 0.13	−0.61 ± 0.11	−0.36 ± 0.77	−0.72 ± 0.13	−0.14 ± 0.03
	168	−1.34 ± 0.85	−0.84 ± 0.21	−7.53 ± 0.08	−0.80 ± 0.07	−1.37 ± 1.06	−0.53 ± 0.19	−1.85 ± 0.22	−0.58 ± 0.05
22	0	0	0	0	0	0	0	0	0
	4	−0.63 ± 0.15	0.08 ± 0.22	−0.55 ± 0.09	−0.06 ± 0.05	−0.23 ± 0.31	−0.18 ± 0.22	−0.38 ± 0.48	0.05 ± 0.34
	24	−1.97 ± 0.26	−0.17 ± 0.18	−7.53 ± 0.08	−0.04 ± 0.15	−1.85 ± 0.06	−0.15 ± 0.16	−0.80 ± 0.15	0.06 ± 0.31
	48	−3.35 ± 0.33	−0.58 ± 0.81	−7.53 ± 0.08	−0.04 ± 0.09	−3.50 ± 0.36	−0.42 ± 0.19	−2.53 ± 0.36	−0.05 ± 0.20
	168	−7.53 ± 0.08	−4.63 ± 2.71	−7.53 ± 0.08	−0.06 ± 0.10	−6.83 ± 0.05	−3.71 ± 1.32	−6.83 ± 0.05	−1.35 ± 1.05

**Table 2:** Microbial inactivation parameters predicted by Weibull model for different temperatures.

	Estimated Parameters			
Temperature (°C)	δ (h)	p	RSS	R^2^
4	1.769	0.222–0.227	0.023	0.982
10	1.567	0.430	0.031	0.970
22	1.509	0.484–0.503	0.001	0.999

Abbreviations: δ and p, survival curves parameters; RSS, residual sum of squares; R^2^, coefficient of determination.

## Data Availability

Data will be made available on request.

## Supplementary Materials

The following supporting information can be downloaded at: https://www.mdpi.com/article/10.3390/nano15120892/s1, Table S1. Hydrodynamic size (nm) and polydispersity index (PDI) for ZnO-SP, ZnO-FL, and ZnO-SH nanoparticles, obtained by DLS. Average and standard deviation (SD) of triplicate analysis. Table S2. Spin-Hamiltonian parameters for [14N] BMPO/OH adduct. Table S3. Antioxidant activity expressed in ascorbic acid equivalent (g L^−1^). Average ± standard deviation. Table S4. Antioxidant activity expressed in Trolox (mg mL^−1^). Average ± standard deviation. Table S5. Stability of dispersions of ZnO-SP, ZnO-FL, and ZnO-SH nanoparticles, obtained by Zeta potential. Average and standard deviation (SD) of triplicate analysis. Figure S1. Original TEM micrographs of ZnO-SP, ZnO-FL, and ZnO-SH. Magnification: 15,000×. Figure S2. Original SEM micrographs of ZnO NPs in contact with *E. coli* after 4 days/22 °C incubation.
