# Supplementary material for: Functional Properties and Safety Considerations of Zinc Oxide Nanoparticles Under Varying Conditions
Source: Nanomaterials (Basel). 2025 Jun 10;15(12):892. doi: 10.3390/nano15120892 (PMC12196094; doi:10.3390/nano15120892)
Supplement: Supplementary file 1 [file nanomaterials-15-00892-s001.zip › nanomaterials-3673355-supplementary.pdf]

Supplementary material

Functional Properties and Safety Considerations of Zinc Oxide Nanoparticles Under Varying Conditions

Ana Rita Mendes [1], Carlos M. Granadeiro [2], Andreia Leite [2], Otmar Geiss [3], Ivana Bianchi [3], Jessica Ponti [3], Dora Mehn [3], Eulália Pereira [2], Paula Teixeira [1] and Fátima Poças [1,4,*]

Table S1. Hydrodynamic size (nm) and polydispersity index (PDI) for ZnO-SP, ZnO-FL, and ZnO-SH nanoparticles, obtained by DLS. Average and standard deviation (SD) of triplicate analysis.

	**ZnO-SP**		**ZnO-FL**		**ZnO-SH**	
Replica	Size (nm)	PDI	Size (nm)	PDI	Size (nm)	PDI
1	310	0.312	1122	0.39	740.3	0.45
2	327	0.302	1230	0.36	824.5	0.48
3	329	0.241	1195	0.38	841.8	0.38
Average	322	0.29	1182	0.38	802	0.44
SD	10	0.04	55	0.01	54	0.05

Table S2. Spin-Hamiltonian parameters for [^{14}N] BMPO/OH adduct.

**Spin adduct**		**giso**	**Hyperfine coupling constant (G)**	
			**aNiso**	**aHiso**
**[^{14}N] BMPO/OH**	ZnO-SP	2.0068	14.20	13.80
	ZnO-FL	2.0067	14.10	13.80
	ZnO-SH	2.0067	14.10	13.80

Table S3. Antioxidant activity expressed in ascorbic acid equivalent (g L^{-1}). Average ± standard deviation.

**Sample**	**Concentration (mg mL^{-1})**	**AAEAC (g L^{-1})**
**ZnO-SP**	0.5	- 0.03 ± 0.01
	1	- 0.03 ± 0.01
**ZnO-FL**	0.5	- 0.04 ± 0.01
	1	- 0.05 ± 0.01
**ZnO-SH**	0.5	- 0.05 ± 0.02
	1	- 0.05 ± 0.01

Table S4. Antioxidant activity expressed in Trolox (mg mL^{-1}). Average ± standard deviation.

Sample	Concentration (mg mL^{-1})	Trolox (mg mL^{-1})
**ZnO-SP**	1	0.00 ± 0.00
	10	- 0.03 ± 0.00
	50	- 0.22 ± 0.02
**ZnO-FL**	1	- 0.02 ± 0.01
	10	- 0.11 ± 0.01
	50	- 0.18 ± 0.12
**ZnO-SH**	1	0.00 ± 0.00
	10	- 0.08 ± 0.00
	50	- 0.28 ± 0.07

Table S5. Stability of dispersions of ZnO-SP, ZnO-FL, and ZnO-SH nanoparticles, obtained by Zeta potential. Average and standard deviation (SD) of triplicate analysis.

**Zeta potential**			
Replica	ZnO-SP	ZnO-FL	ZnO-FL
1	-43.3	-50.8	-56.5
2	-43.7	-50.8	-57.3
3	-44.1	-50.6	-58.6
Average	-43.7	-50.7	-57.5
SD	0.4	0.1	1.1

Original microscopy images

Transmission electron microscopy:

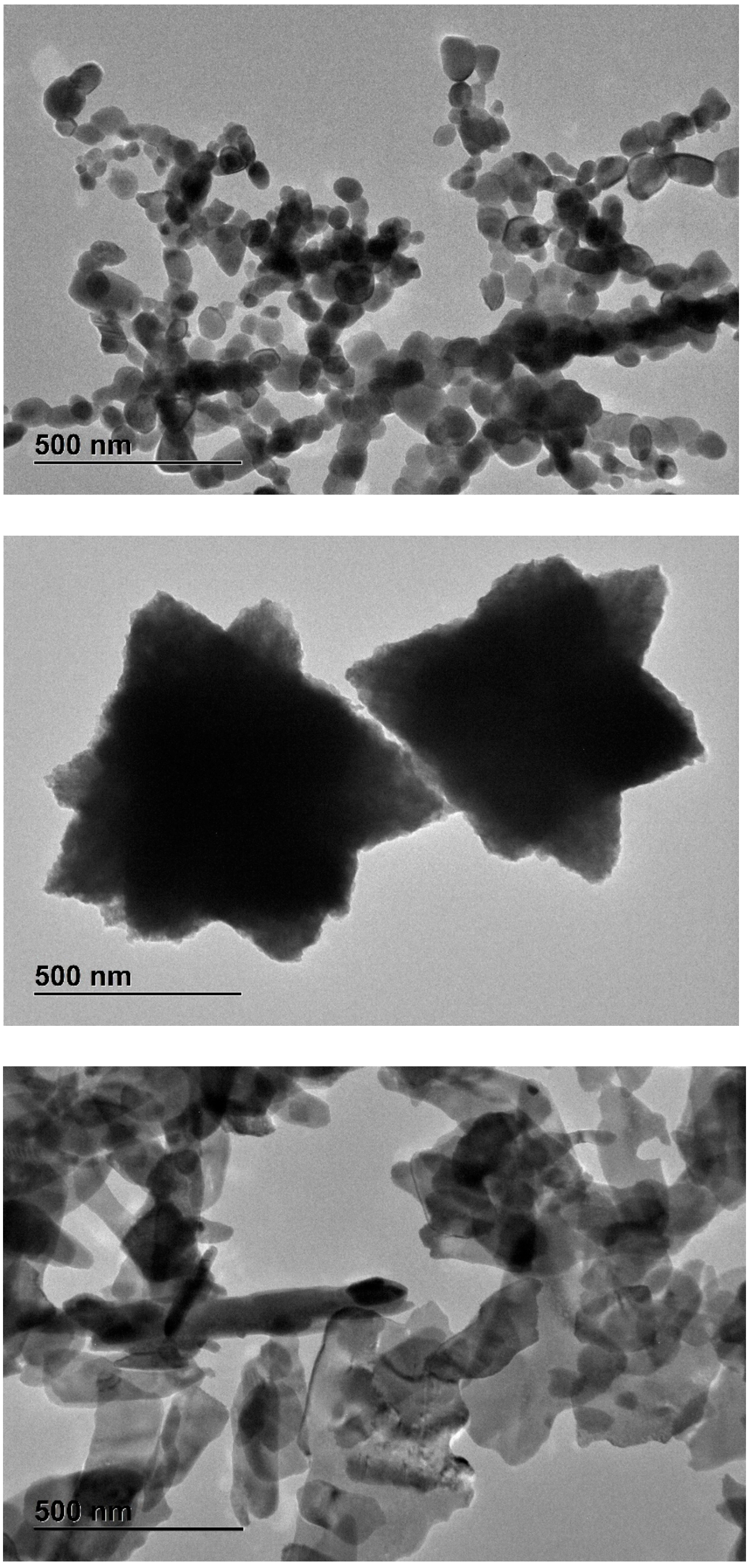

Figure S1. Original TEM micrographs of ZnO-SP, ZnO-FL, and ZnO-SH. Magnification: 15 000x.

Scanning electron microscope:

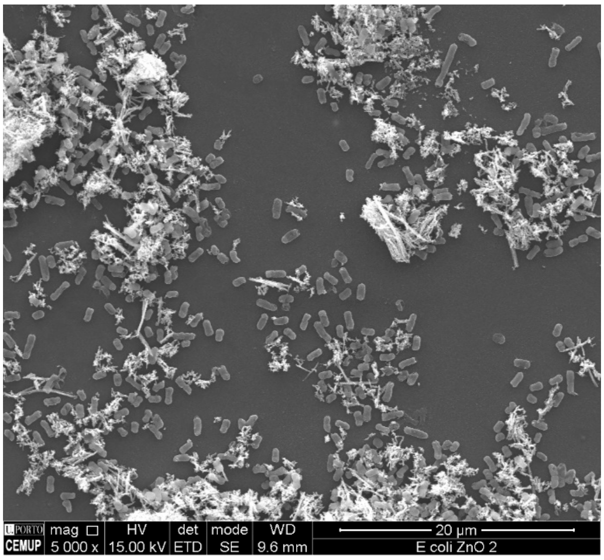

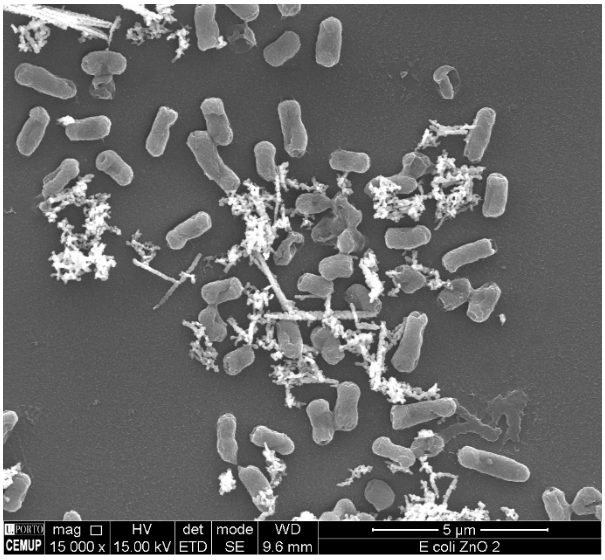

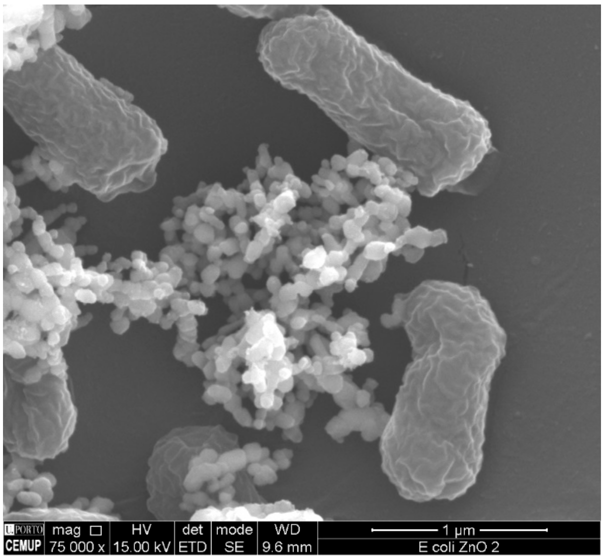

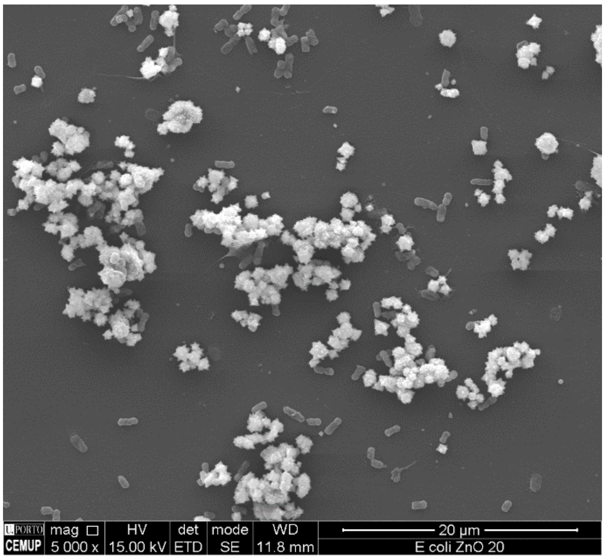

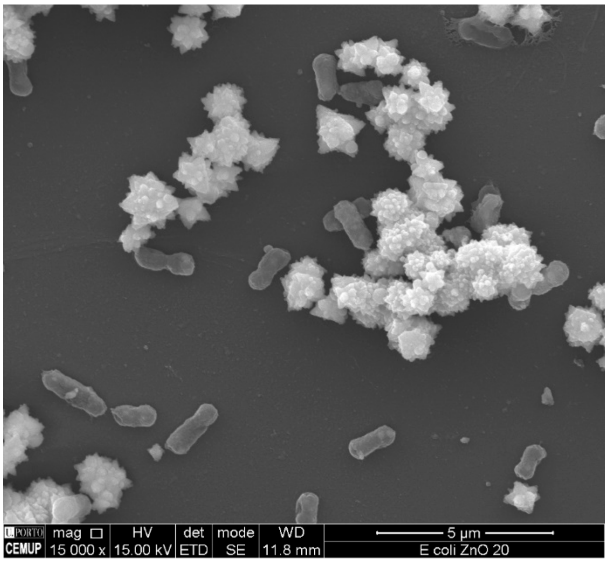

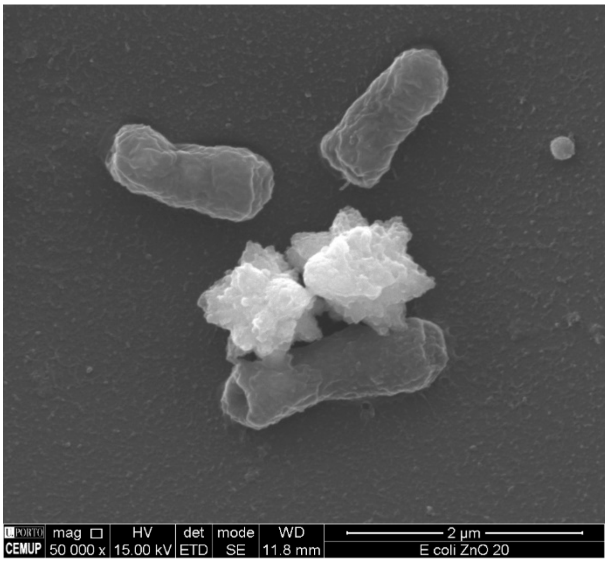

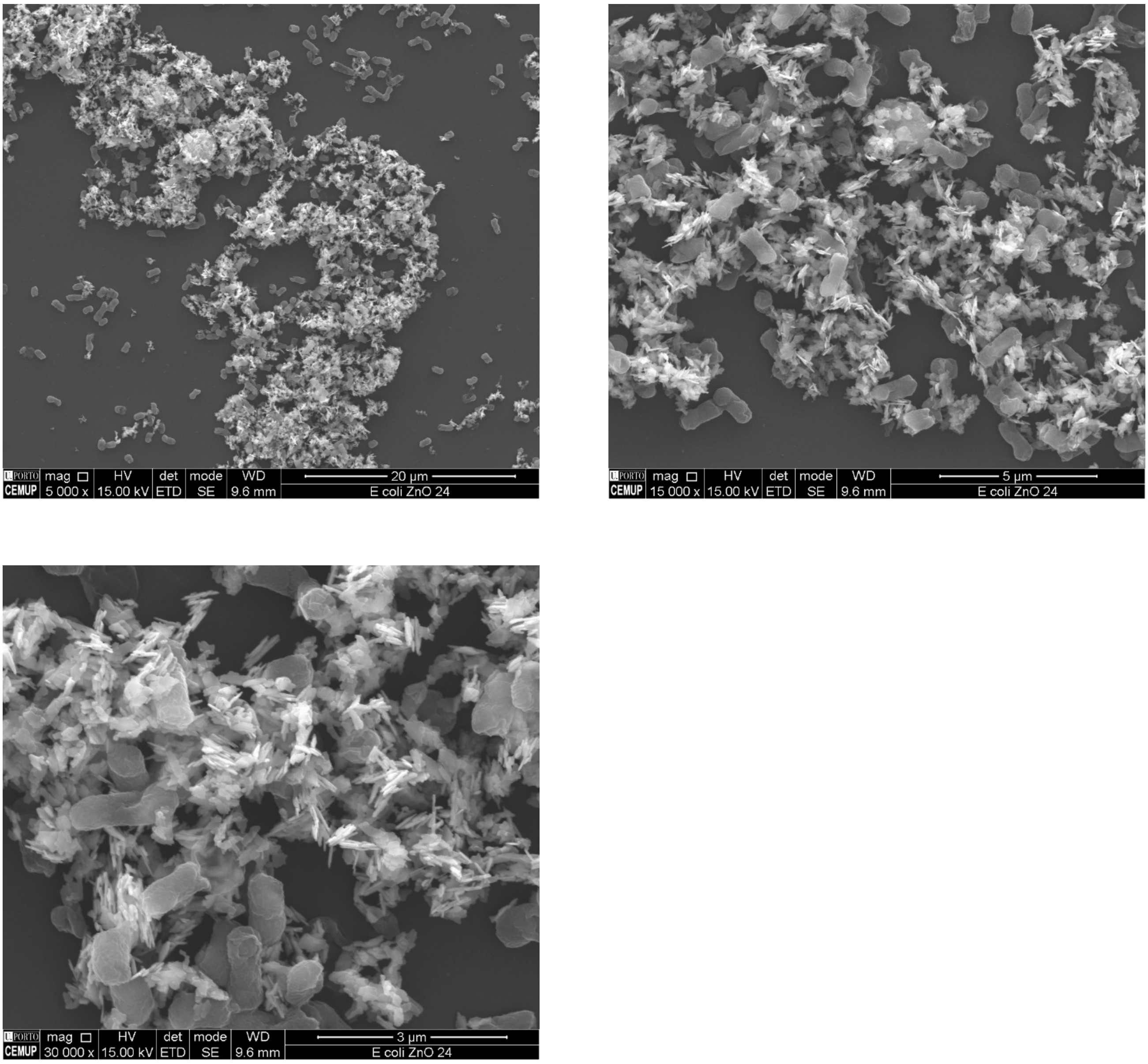

Figure S2. Original SEM micrographs of ZnO NPs in contact with *E. coli* after 4 days/ 22 °C incubation.
